# Ablation and antiarrhythmic drug effects on *PITX2*^+/−^ deficient atrial fibrillation: A computational modeling study

**DOI:** 10.3389/fcvm.2022.942998

**Published:** 2022-07-19

**Authors:** Ze Jin, Inseok Hwang, Byounghyun Lim, Oh-Seok Kwon, Je-Wook Park, Hee-Tae Yu, Tae-Hoon Kim, Boyoung Joung, Moon-Hyoung Lee, Hui-Nam Pak

**Affiliations:** Yonsei University College of Medicine, Yonsei University Health System, Seoul, South Korea

**Keywords:** atrial fibrillation, computational modeling, *PITX2*, dominant frequency, antiarrhythmic drug

## Abstract

**Introduction:**

Atrial fibrillation (AF) is a heritable disease, and the paired-like homeodomain transcription factor 2 (*PITX2*) gene is highly associated with AF. We explored the differences in the circumferential pulmonary vein isolation (CPVI), which is the cornerstone procedure for AF catheter ablation, additional high dominant frequency (DF) site ablation, and antiarrhythmic drug (AAD) effects according to the patient genotype (wild-type and *PITX2*^+/−^ deficient) using computational modeling.

**Methods:**

We included 25 patients with AF (68% men, 59.8 ± 9.8 years of age, 32% paroxysmal AF) who underwent AF catheter ablation to develop a realistic computational AF model. The ion currents for baseline AF and the amiodarone, dronedarone, and flecainide AADs according to the patient genotype (wild type and *PITX2*^+/−^ deficient) were defined by relevant publications. We tested the virtual CPVI (V-CPVI) with and without DF ablation (±DFA) and three virtual AADs (V-AADs, amiodarone, dronedarone, and flecainide) and evaluated the AF defragmentation rates (AF termination or changes to regular atrial tachycardia (AT), DF, and maximal slope of the action potential duration restitution curves (Smax), which indicates the vulnerability of wave-breaks.

**Results:**

At the baseline AF, mean DF (*p* = 0.003), and Smax (*p* < 0.001) were significantly lower in *PITX2*^+/−^ deficient patients than wild-type patients. In the overall AF episodes, V-CPVI (±DFA) resulted in a higher AF defragmentation relative to V-AADs (65 vs. 42%, *p* < 0.001) without changing the DF or Smax. Although a *PITX2*^+/−^ deficiency did not affect the AF defragmentation rate after the V-CPVI (±DFA), V-AADs had a higher AF defragmentation rate (*p* = 0.014), lower DF (*p* < 0.001), and lower Smax (*p* = 0.001) in *PITX2*^+/−^ deficient AF than in wild-type patients. In the clinical setting, the *PITX2*^+/−^ genetic risk score did not affect the AF ablation rhythm outcome (Log-rank *p* = 0.273).

**Conclusion:**

Consistent with previous clinical studies, the V-CPVI had effective anti-AF effects regardless of the *PITX2* genotype, whereas V-AADs exhibited more significant defragmentation or wave-dynamic change in the *PITX2*^+/−^ deficient patients.

## Graphical Abstract

AF, atrial fibrillation; ECG, electrocardiogram; CPVI, complete pulmonary vein isolation; AAD, antiarrhythmic drug; AT, atrial tachycardia. 
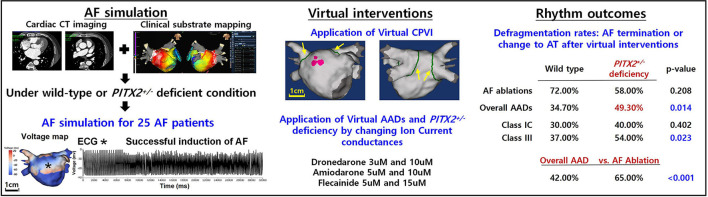

## Main discoveries

- Compared with wild-type, the *PITX2*^+/−^ deficient AF model exhibited different electrophysiology and AF wave dynamics.
- Ablation resulted in a higher AF defragmentation rate than AADs in human AF computational modeling.
- AF defragmentation rate did not differ depending on the *PITX2* genotype after a virtual AF ablation.
- Virtual AADs exhibited more significant defragmentation in the *PITX2*^+/−^ deficient genotype with a lower mean DF and Smax than wild type.

## Introduction

Atrial fibrillation (AF) is a common arrhythmia disease with a prevalence of 1.7% in the Korean population. AF numbers are expected to increase; therefore, AF is considered a major health care issue in Korea (1). Recently, the EAST-AFNET4 trial demonstrated that active AF rhythm control reduced morbidity and mortality risk (2). Multiple randomized clinical trials documented the superior efficacy of AF rhythm control by AF catheter ablation (AFCA) relative to the treatment with antiarrhythmic drugs (AADs) (3). Nevertheless, about 40% of patients with AF achieve effective rhythm control with AADs (4).

Atrial fibrillation is a heritable disease, and the paired-like homeodomain transcription factor 2 (*PITX2*) gene is highly associated with AF (5). Several clinical studies have reported the difference in the efficacy of AF rhythm control treatment according to the *PITX2* genotype, but those were small retrospective studies, and the underlying mechanism is still not understood (6–13). AF computational modeling is useful for AF mechanism research, which is difficult to reveal through clinical or experimental studies (14). With recent improvements in computational technology and power, sophisticated AF computation modeling has become possible. Virtual ablation or virtual AAD responses can be tested on a virtual twin that reflects the anatomy, fibrosis, fiber orientation, and electrophysiological characteristics of patients with AF, and the wave dynamics generated from hundreds of thousands of nodes can also be evaluated (15–18).

In this study, we explored the response and mechanism of AFCA and AADs according to the patient *PITX2* genotype. We used AF computational modeling integrated with clinical electroanatomical maps of 25 AAD-resistant or intolerable patients with AF who underwent AFCA, and the effects of various virtual interventions (AFCA and three different AADs) attempted under the same conditions were compared and evaluated. The ion currents associated with baseline AF and AADs (amiodarone, dronedarone, and flecainide) according to the genotype (wild type and *PITX2*^+/−^ deficient) were defined by the relevant publications.

## Methods

### Ethical approval

This study protocol adhered to the Declaration of Helsinki and was approved by the Institutional Review Board of Severance Cardiovascular Hospital, Yonsei University Health System. All patients included in the Yonsei AF Ablation Cohort Database (ClinicalTrials.gov Identifier: NCT02138695) provided written informed consent for use of their clinical data for computational modeling studies.

### A 3D computational model of the left atrium

**Figure 1:**
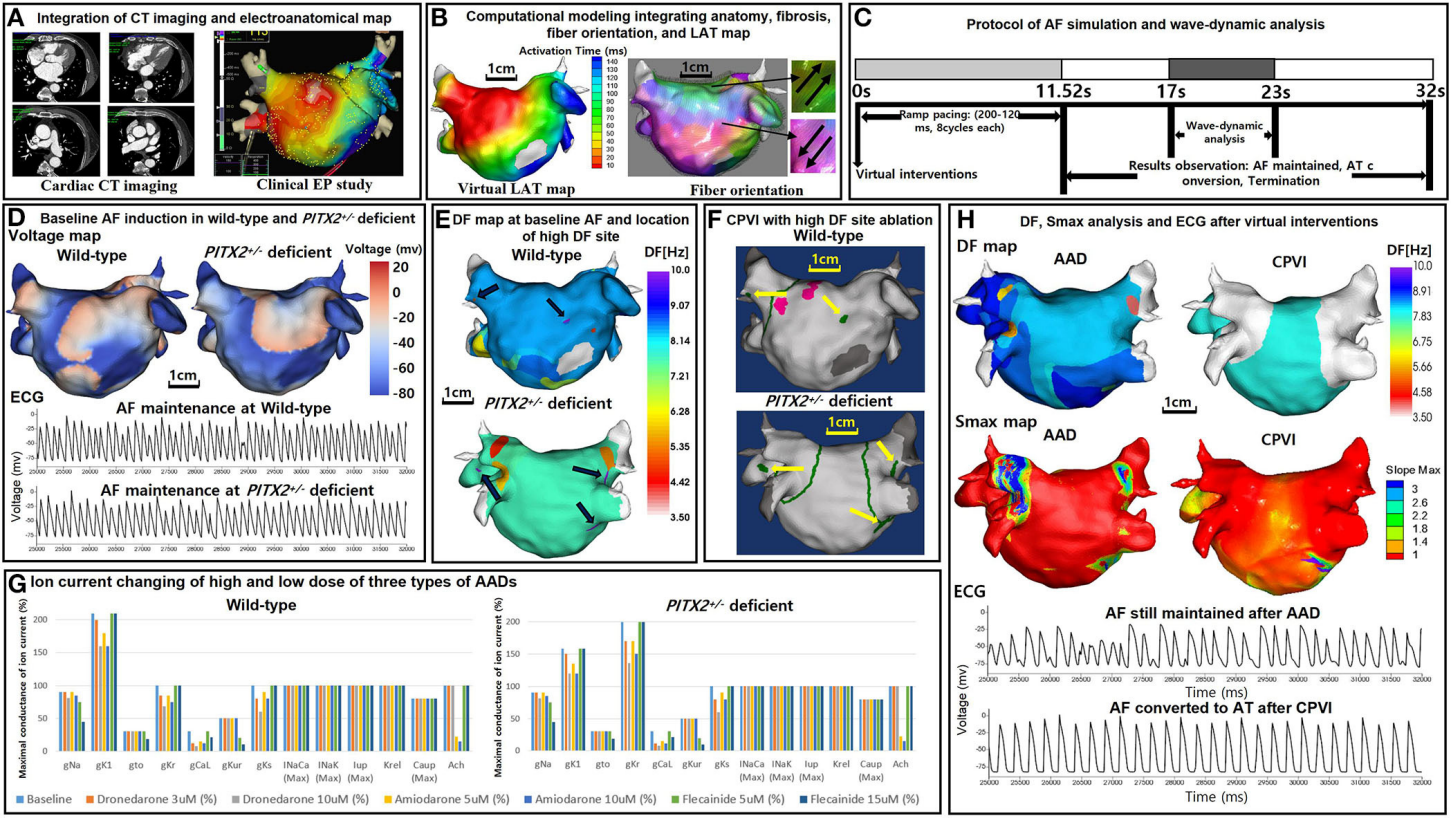
Study protocol of the computational atrial modeling, AF simulation, and virtual interventions. **(A)** Integration of the CT imaging and electroanatomical map. **(B)** Computational modeling integrating the anatomy, fibrosis, fiber orientation, and LAT map. **(C)** Protocol of the AF simulation and wave-dynamic analysis. AF was induced in each case using AF pacing from 200 to 120 ms with eight beats per cycle lasting a total of 11,520 ms based on the wild-type *PITX*2^+/−^ deficient AF baseline ion current settings. AF maintenance was observed for 20,480 ms after induction (overall 32 s including pacing), and the wave dynamics of the DF and Smax were analyzed from 17,000 to 23,000 ms. **(D)** Baseline AF induction under wild-type and *PITX*2^+/−^ deficient backgrounds. The voltage maps and ECGs indicate a successful AF induction during the wild-type and *PITX*2^+/−^ deficient baselines. **(E)** 3D DF map of the baseline AF under wild-type and *PITX*2^+/−^ deficient backgrounds. The black arrows indicate the locations of the high DF sites on the 3D DF map. **(F)** Virtual CPVI with a high DF site ablation. The green lines indicate the CPVI and yellow arrows indicate the ablated regions of the high DF sites. The pink sites indicate the pacing site. **(G)** Ion current changes with the high and low doses of the three types of AADs. Ion current changes with the high and low doses of the three types of AADs under the wild-type and *PITX*2^+/−^ deficient backgrounds. For *PITX*2^+/−^ deficiency, the I_K1_ decreased by 25% and the I_Kr_ increased by 100% as compared to that with the wild-type status, while the other ion currents remain the same as the wild-type. **(H)** Smax and DF analysis after AADs and the CPVI. The ECGs indicate AF was maintained after AADs, and AF converted to AT after the CPVI. CT, computed tomography; EP, electrophysiology; LAT, local activation time; *PITX2*, paired-like homeodomain transcription factor 2; CPVI, complete pulmonary vein isolation; DF, dominant frequency; Smax, the Maximal slope of the restitution curves; AF, atrial fibrillation; AT, atrial tachycardia; ECG, electrocardiogram; AAD, antiarrhythmic drug.

Figures 1A,B illustrate the protocol for computational atrial modeling. To obtain the clinical electroanatomical data, we collected the bipolar electrogram data on the LA surface to produce clinical voltage data of 25 patients who underwent AFCA. The interpolated voltage data were generated from bipolar electrograms recorded from >500 points on the atrial surface using a circular mapping catheter and CT images (Figure 1A). The coordinates of the electroanatomical map (NavX, Abbott, Inc., Chicago, IL, USA; CARTO System, Biosense Webster, Diamond Bar, CA, USA) were precisely aligned with patient clinical heart CT images, followed by registration between the electroanatomical maps and clinical CT data (Figure 1A).

To reflect the tissue characteristics in the 3D left atrium (LA) model, we performed electroanatomical modeling and fibrosis and fiber orientation modeling. Electroanatomical modeling combining personalized CT images with the clinical voltage data was used to obtain a personalized 3D LA model of each patient. The surface of the 3D LA model was composed of triangular meshes containing 400,000–500,000 geometric elements, and the mean distance between the adjacent elements was 235.1 ± 32.1 μm. Interpolation of the clinical voltage data was used to create the virtual voltage data. We used the inverse distance weighting method (19) to represent the interpolation of the electroanatomical map values during the simulation procedures.

Integrating the electroanatomical maps containing the clinical voltage data and 3D LA maps onto the CT-based mesh models was conducted over four steps: geometry, trimming, field scaling, and alignment (15). The geometry was generated during the electroanatomical map creation using a catheter. After the geometry step, unnecessary artifact was removed, and the ostial position was used for the separation of the LA appendage and pulmonary vein (PV) regions during the trimming step. The field scaling step indicated the optimal scaling of the inter-electrode spacing and CT images. Lastly, the alignment step involved the registration of the alignment points through a coordinate transformation using an accurately defined ostium, along with the integration of CT images and anatomical maps. We used the Courtemanche-Ramirez-Nattel model (20–22) for the wild-type sinus rhythm (SR) status. All ion currents for the wild-type SR status were set to 100%. For the wild-type AF atrial ionic remodeling, the sodium current (I_Na_), transient outward potassium current (I_to_), L-type calcium current (I_CaL_), ultrarapid outward current (I_Kur_), and calcium current concentration in the uptake compartment (I_Caup_) decreased by 10, 70, 70, 50, and 20% respectively, and the inwardly rectifying potassium current (I_K1_) increased by 110% as compared to the Courtemanche-Ramirez-Nattel model (23).

We simulated the clinical local activation data using the 3D LA model, which reflected the cardiac structural and fiber orientation (Figure 1B). To achieve each personalized virtual LA model, synchronization of the clinical local activation time (LAT) map and the virtual LAT map was performed (Figure 1B). The virtual LAT map diffusion coefficient was adjusted to accurately match the conduction velocity (CV) of the clinical LAT map (15). Bipolar voltage data obtained from catheter ablation mapping were matched onto the computational nodes of the 3D LA model, and the fibrotic area locations were determined using the map (Figure 1B). The fibrosis status of each node was numerically defined and determined using the relationship between the probability of fibrosis and bipolar voltage (24, 25). The fiber orientation was defined in the meshes of each patient geometry and adjusted based on the clinical local activation time map (26, 27). Parallel tasking was used for the fiber tracking step and a visual display of the fiber orientation onto the 3D LA map was conducted during the visualization step (Figure 1B). For the ion currents of the fibrotic cells, the I_K1_, I_CaL_, and I_Na_ were decreased by 50, 50, and 40%, respectively, as compared to normal cells (25). The conductivity of the model was based on the status and shape of the fibrosis (25). The reaction-diffusion equation for the cardiac wave propagation was solved numerically and adjusted based on the specific conduction velocity in each case to represent personalized AF simulations (23).

### *PITX2^+/−^* deficient incorporation

The Syeda et al. model (13) was used for the *PITX*2^+/−^ deficiency status. The I_K1_ was decreased by 25% and the rapidly activating delayed rectifying potassium current (I_Kr_) was increased by 100% as compared to the wild-type status. Therefore, for the *PITX*2^+/−^ deficiency AF baseline status, the I_Na_, I_to_, I_CaL_, I_Kur_, I_Caup_ were decreased by 10, 70, 70, 50, 20%, respectively, whereas the I_K1_ and I_Kr_, were increased by 58 and 100%, respectively, as compared to the Courtemanche-Ramirez-Nattel model.

### AF simulation

Our graphical user interface software (Model:SH01, CUVIA; Laonmed Inc., Seoul, Korea) integrated the fibrosis formation and fiber orientation into the LA surface and enabled virtual AF induction and AF wave-dynamic changes (28). Figure 1C shows the process used in the study protocol. We induced AF in each case using AF pacing from 200 to 120 ms with eight beats per cycle lasting a total of 11,520 ms, based on the appropriate ion current settings. Each virtual pacing location corresponded to the clinical activation time map for realistic LA modeling, and the pacing sites were matched precisely to reflect each personalized LA model. AF maintenance was observed for 20.48 s after the induction (overall 32 s including pacing). Figure 1D indicates the successful AF induction during the baseline status under wild type and *PITX2*^+/−^ deficient backgrounds. We defined a successful AF induction according to the electrograms in each LA model, and AF defragmentation involved AF termination and AF conversion to atrial tachycardia (AT).

### Virtual ablations

We applied virtual ablation and virtual AADs to our realistic AF model. For the virtual ablation, the membrane potential of the ablated regions was set at zero to produce a permanent conduction block interrupting the cardiac wave propagation. First, we performed a virtual circumferential pulmonary vein isolation (CPVI; V-CPVI). Under conditions of a CPVI alone, we initiated the AF induction as described in Figure 1C under wild type and *PITX*2^+/−^ deficient backgrounds. Then, we applied a virtual high dominant frequency (DF) site ablation to failed AF defragmentation episodes after the CPVI alone and initiated AF induction again. High DF sites were targeted based on the 3D DF map during baseline AF under wild-type and *PITX*2^+/−^ deficient backgrounds (Figures 1E,F).

### Virtual AADs

Three types of AADs were used for the study: amiodarone, dronedarone, and flecainide. We tested the high and low dose effects of each AAD; 5 and 10 μM amiodarone, 3 and 10 μM dronedarone, and 5 and 15 μM flecainide. All ionic changes for each drug were derived from previously reported references (Supplementary Table 3) and the AADs were designed by changing them relative to the AF baseline model under wild-type or *PITX*2^+/−^ deficient backgrounds (Figure 1G). The degree of change in the value varied within each AAD to resemble low and high dosage. Supplementary Table 2 shows the complete list of ion currents for the baseline AF status and AADs under wild type and *PITX*2^+/−^ deficient backgrounds, and the references for each AAD ion current setting are listed in Supplementary Table 3.

### Analysis of the spatial changes in the AF wave-dynamics

Our graphic processing unit (GPU)-based customized software (CUVIA, Model: SH01; Laonmed Inc., Seoul, Korea) was used virtually to define the ablated regions and apply appropriate ion current settings for the baseline AF and AADs. The DF and Smax were analyzed using this same GPU-based software (Figure 1H). During baseline AF, we additionally analyzed the action potential duration at 90% repolarization (APD_90_), conduction velocity (CV), and AF cycle length. A pacing cycle length of 600 ms was used to measure the APD_90_ (29) and CV. The region of interest for the APD_90_ and CV was from the LA high septum (pacing sites) to the LA appendage. The action potential duration (90%) was measured in the single-cell environment. However, at the tissue level, the APD_90_ values were heterogeneous among patients due to electroanatomical characteristics and LA tissue curvature (29). The APD_90_ and CV were measured using the SR ion currents while the mean Smax, DF, and AFCL were calculated using AF ion currents.

### Statistical analysis

Categorical variables are reported as numbers (percentages). To investigate the normal distribution, continuous variables were tested using the Shapiro-Wilk or Kolmogorov-Smirnov tests. Continuous variables without a normal distribution are expressed as medians with interquartile range (IQR), while those with a normal distribution are expressed as means ± standard deviations. The proportion of categorical variables was compared among the groups using a Chi-square or Fisher's exact test. Continuous variables without a normal distribution were analyzed using the Mann-Whitney U test between two groups and the Kruskal-Wallis test among three groups. Continuous variables with a normal distribution were tested using ANOVA tests among three groups. A *p*-value <0.05 was considered statistically significant. All statistical analyses were performed using SPSS (Statistical Package for Social Sciences, Chicago, IL, USA) software for Windows (version 26).

## Results

### Characteristics of *PITX2^+/−^* deficient AF

**Figure 2:**
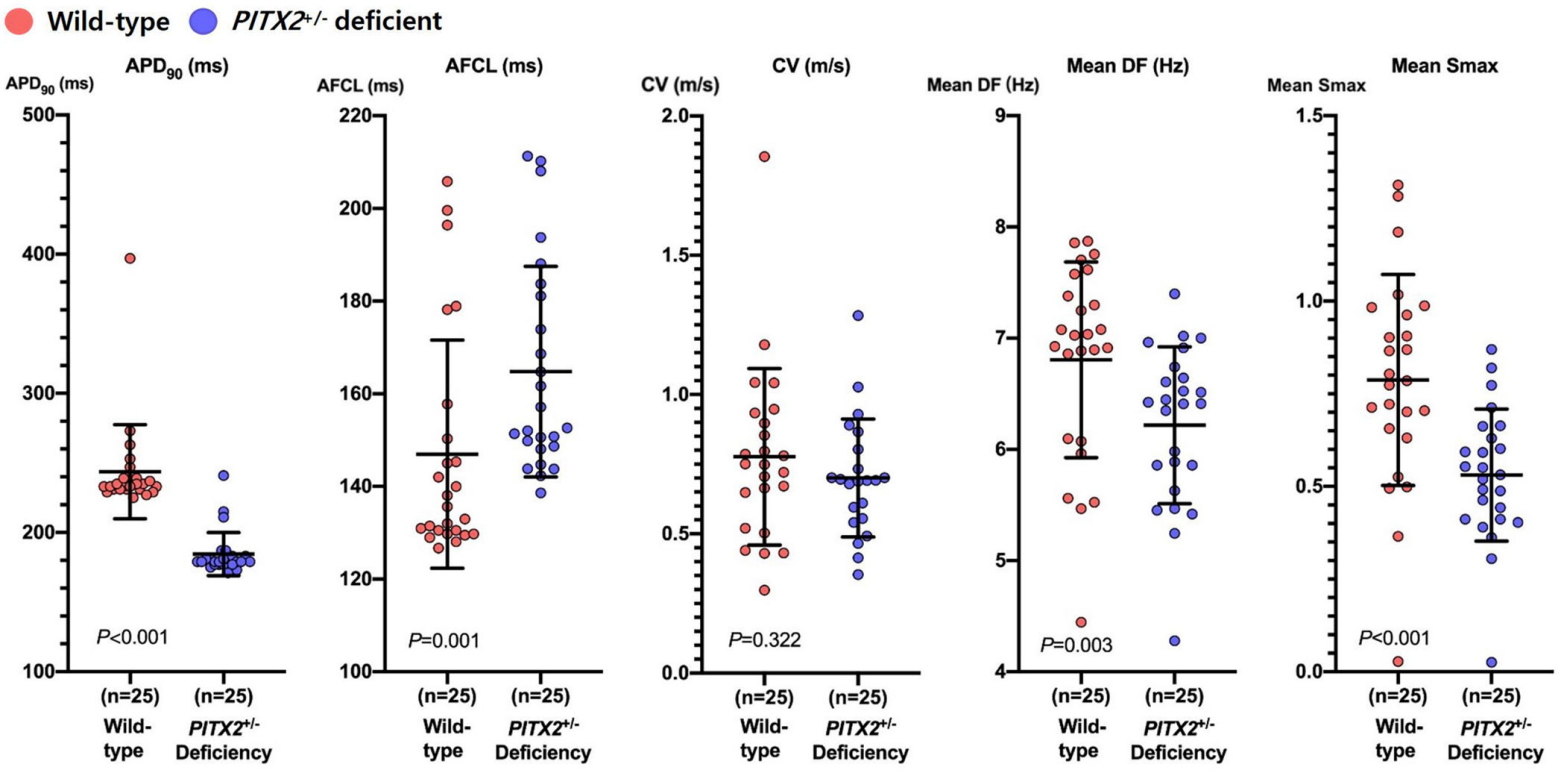
Characteristics of wild-type and *PITX2*^+/−^ deficient baseline AF. Genotype-dependent comparisons of the APD_90_, CV, mean Smax, mean DF, and AFCL depend on the baseline AF. Every group includes an identical number of samples (*n* = 25). APD_90_, action potential duration 90%; CV, Conduction velocity; Smax, the Maximal slope of the restitution curves; AFCL, AF cycle length; DF, Dominant frequency; *PITX2*, paired-like homeodomain transcription factor 2.

We applied two different genotypes (wild type and *PITX2*^+/−^ deficient) to the realistic AF computational modeling of 25 patients who underwent AFCA (68% men, 59.8 ± 9.8 years of age, 32% paroxysmal AF; Supplementary Table 1). We evaluated the effects of two different ablation protocols (CPVI and CPVI+DF ablation) and two different doses of three AADs (amiodarone, dronedarone, and flecainide). After measuring the APD_90_, we induced AF by virtual ramp pacing and there was no AF defragmentation of the baseline AF during the 32 s waiting period. In the *PITX2*^+/−^ deficient AF condition, the APD_90_ was shorter (233 ms [231, 240] to 179 ms [177, 183], *p* < 0.001), AF cycle length longer (135.62 ms [130.13, 154.04] to 152.62 ms [148.36, 182.41], *p* = 0.001), DF (7.025 Hz [6.085, 7.478] to 6.411 Hz [5.744, 6.693], *p* = 0.003) and Smax (0.785 [.644,0.973] to.531 [.411,0.646], *p* < 0.001) significantly lower than that in the wild-type AF condition (Figure 2).

### Anti-AF effects of virtual ablation and AADs

**Figure 3:**
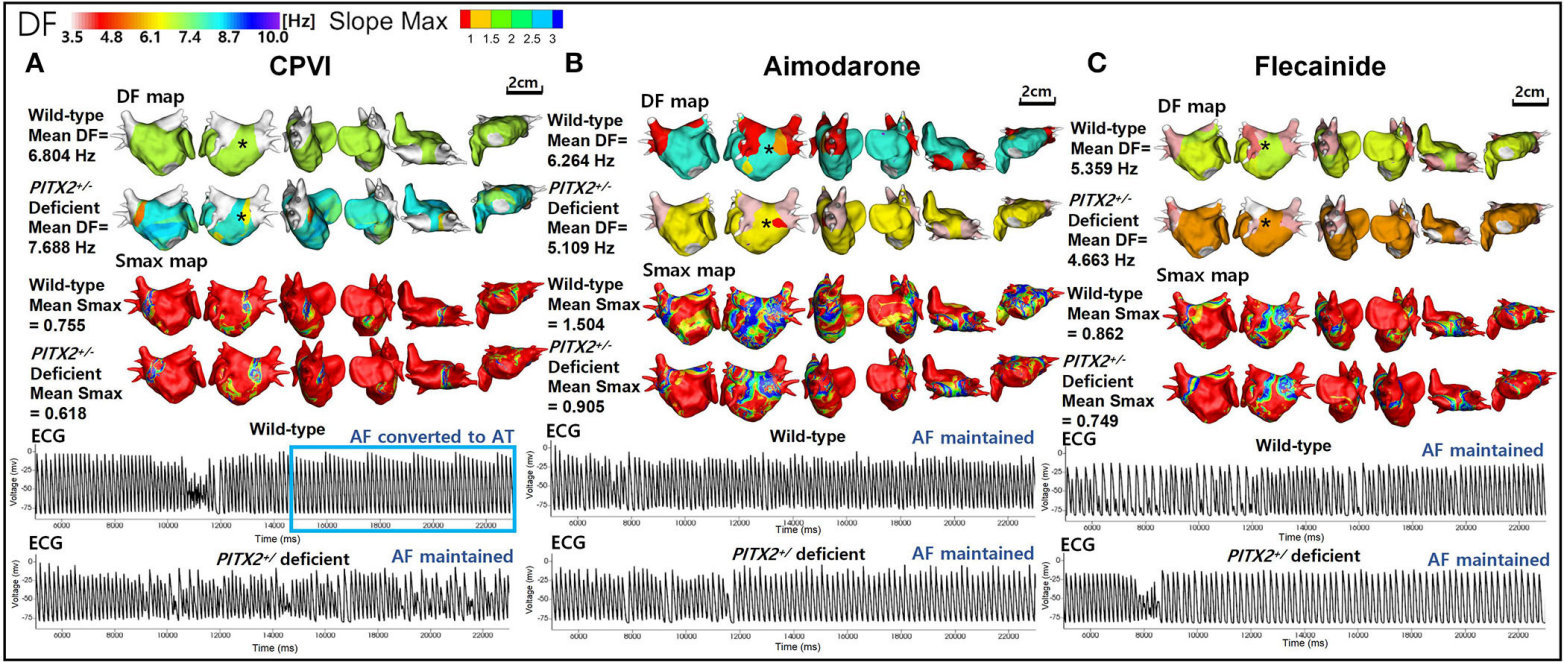
Wave-dynamic change after a virtual CPVI and AADs. **(A)** The ECGs were obtained at the black ^*^ sites in the DF maps and indicate that AF converted to AT after the CPVI during a wild-type condition was still maintained during a *PITX*2^+/−^ deficient condition. **(B)** The ECGs were obtained at the black ^*^ sites in the 3D DF maps and indicate that AF was still maintained after high dose amiodarone under both wild-type and *PITX*2^+/−^ deficient backgrounds. **(C)** The ECGs were obtained at the black ^*^ sites in the DF maps and indicate that the AF was still maintained after high dose flecainide under both wild-type and *PITX*2^+/−^ deficient backgrounds. DF, dominant frequency; Smax, the maximal slope of the restitution curves; CPVI, complete pulmonary vein isolation; AF, atrial fibrillation; AT, atrial tachycardia; ECG, electrocardiogram; *PITX2*, paired-like homeodomain transcription factor 2.

Table 1 summarizes the AF defragmentation or termination rates and wave-dynamics changes after 100 virtual ablations (CPVI with or without DF ablation) and 300 virtual AAD interventions. Overall interventions including a CPVI ± DF ablation and AADs significantly increased the AF termination (22.3%, *p* < 0.001) and defragmentation (47.8%) rates as compared to the baseline AF (0%). When we compared the overall virtual interventions and overall AADs, CPVI±DF ablations resulted in a significantly higher AF defragmentation rate than AADs (65 vs. 42%, *p* < 0.001, Figure 3) without changing the DF or Smax (Table 1). In contrast, AADs significantly reduced the mean DF (6.625 Hz [5.88, 7.045] to 5.903 Hz [5.109, 6.388], *p* < 0.001). There were no significant differences in the AF defragmentation or termination rates, or the DF or Smax between the CPVI and CPVI+DF ablation or among amiodarone, dronedarone, and flecainide (Table 1).

**Table 1:** Defragmentation rate and wave-dynamic changes in the overall AF episodes (Wild type and *PITX2*^+/−^ deficient).

	**AF defragmentation, %(n)**	**AF termination, %(n)**	**Mean DF, (Hz)**	**Mean Smax**
Baseline (*n =* 50)	0% (0/50)	0% (0/50)	6.625 [5.880, 7.045]	0.644 [0.491, 0.831]
CPVI(±DFA) (*n =* 100)	65.0% (65/100)	25.0% (25/100)	6.788 [5.200, 7.688]	0.739 [0.519, 1.030]
Overall AADs (*n =* 300)	42.0% (126/300)	21.3% (64/300)	5.903 [5.109, 6.388]	0.739 [0.537, 0.996]
p-value	<0.001	0.488	<0.001	0.958
CPVI	58.0% (29/50) ^*^	24.0% (12/50)	7.138	0.798
(*n =* 50)			[5.349, 7.716]	[0.523, 1.073]
CPVI+DF ablation (*n =* 50)	72.0% (36/50)	26.0% (13/50)	6.343 [5.200, 7.484]	0.696 [0.515, 0.975]
p-value	0.208	1	0.344	0.291
Amiodarone (*n =* 100)	45.0% (45/100)	23.0% (23/100)	5.886 [5.089, 6.351]	0.803 [0.591, 1.053]
Dronedarone (*n =* 100)	46.0% (46/100)	22.0% (22/100)	6.062 [5.250, 6.727]	0.661 [0.510, 0.975]
Flecainide (*n =* 100)	35.0% (35/100)	19.0% (19/100)	5.818 [5.187, 6.290]	0.738 [0.524, 0.939]
p-value	0.239	0.822	0.234	0.075

*AAD, antiarrhythmic drug; AF, atrial fibrillation; DF, dominant frequency; Smax, the maximal slope of action potential duration restitution curves; CPVI, complete pulmonary vein isolation*.

* *p = 0.045 vs. overall AADs*.

### *PITX2^+/−^* genotype-dependent responsiveness to anti-AF interventions

We summarize the *PITX2*^+/−^ genotype-dependent changes after a virtual intervention or AADs in Table 2. Overall, the virtual ablation (72%, *p* < 0.001) or CPVI alone (68%, *p* = 0.003) exhibited better AF defragmentation rates than the overall AADs (34.7%) in the wild-type AF, but not the *PITX*2^+/−^ deficient AF. Virtual ablation did not exhibit any difference in the defragmentation rate (*p* = 0.208) or changes in the DF (*p* = 0.965) depending on the genotype but resulted in a lower Smax in the *PITX2*^+/−^ deficient genotype than wild-type control (*p* = 0.023). After the overall AADs, *PITX*2^+/−^ deficient AF was more easily defragmented (49.3 vs. 34.7%, *p* = 0.014) and had a greater significant reduction in the mean DF (*p* < 0.001) and mean Smax (*p* = 0.001) as compared to the wild type (Table 2; Figure 3).

**Table 2:** Defragmentation rate and wave-dynamic changes after virtual interventions according to the genotype.

	**AF defragmentation, %(*n*)**		***p*-value**	**AF termination, %(*n*)**		***p*-value**	**Mean DF, (Hz)**		***p*-value**	**Mean Smax**		***p*-value**
	**Wild type**	***PITX*2^+/−^Deficiency**		**Wild type**	***PITX*2^+/−^Deficiency**		**Wild type**	***PITX*2^+/−^Deficiency**		**Wild type**	***PITX*2^+/−^Deficiency**	
Baseline AF (*n =* 25)	0% (0/25)	0% (0/25)	NA	0% (0/25)	0% (0/25)	NA	7.025 [6.085, 7.478]	6.411 [5.744, 6.693]	0.003	0.785 [0.644, 0.973]	0.531 [0.411, 0.646]	<0.001
CPVI(±DFA)	72.0%	58.0%	0.208	28.0%	22.0%	0.645	6.595	6.788	0.965	0.831	0.704	0.023
(*n =* 50)	(36/50)	(29/50)		(14/50)	(11/50)		[5.084, 7.820]	[5.202, 7.685]		[0.566, 1.143]	[0.403, 0.965]	
Overall AADs (*n =* 150)	34.70% (52/150)	49.30% (74/150)	0.014	23.30% (35/150)	19.30% (29/150)	0.481	6.188 [5.584, 6.766]	5.481 [4.920, 6.040]	<0.001	0.799 [0.582, 1.100]	0.668 [0.474, 0.910]	0.001
*p*-value	<0.001	0.329	NA	0.57	0.839	NA	0.143	<0.001	NA	0.729	0.729	NA
CPVI (*n =* 25)	68.0%^*^ (17/25)	48.0% (12/25)	0.252	28.0% (7/25)	20.0% (5/25)	0.742	6.804 [4.969, 7.965]	7.192 [6.223, 7.688]	0.897	0.956 [0.582, 1.272]	0.739 [0.465, 0.973]	0.067
CPVI+DF ablation	76.0%	68.0%	0.754	28.0%	24.0%	1	6.386	6.225	0.976	0.75	0.676	0.16
(*n =* 25)	(19/25)	(17/25)		(7/25)	(6/25)		[4.969, 7.476]	[5.201, 7.615]		[0.560, 1.087]	[0.369, 0.927]	
*p*-value	0.754	0.252	NA	1	1	NA	0.617	0.402	NA	0.362	0.449	NA
Amiodarone (*n =* 50)	38.0% (19/50)	52.0% (26/50)	0.228	22.0% (11/50)	24.0% (12/50)	1	6.188 [5.321, 6.749]	5.457 [4.920, 5.988]	0.001	0.926 [0.682, 1.208]	0.681 [0.477, 0.914]	0.001
Dronedarone (*n =* 50)	36.0% (18/50)	56.0% (28/50)	0.07	28.0% (14/50)	16.0% (8/50)	0.227	6.358 [5.558, 7.141]	5.445 [5.100, 6.341]	0.003	0.724 [0.534, 1.049]	0.626 [0.474, 0.950]	0.138
Flecainide (*n =* 50)	30.0% (15/50)	40.0% (20/50)	0.402	20.0% (10/50)	22.50% (9/50)	1	6.06 [5.634, 6.627]	5.596 [4.758, 5.910]	<0.001	0.743 [0.574, 0.993]	0.713 [0.444, 0.846]	0.16
p-value	0.748	0.253	NA	0.694	0.658	NA	0.279	0.504	NA	0.037	0.781	NA

*AF, atrial fibrillation; DF, dominant frequency; Smax, the maximal slope of action potential duration restitution curves; CPVI, complete pulmonary vein isolation; AAD, antiarrhythmia drug; PITX2, paired-like homeodomain transcription factor 2*.

* *p = 0.003 vs. wild-type overall AADs*.

**Figure 4:**
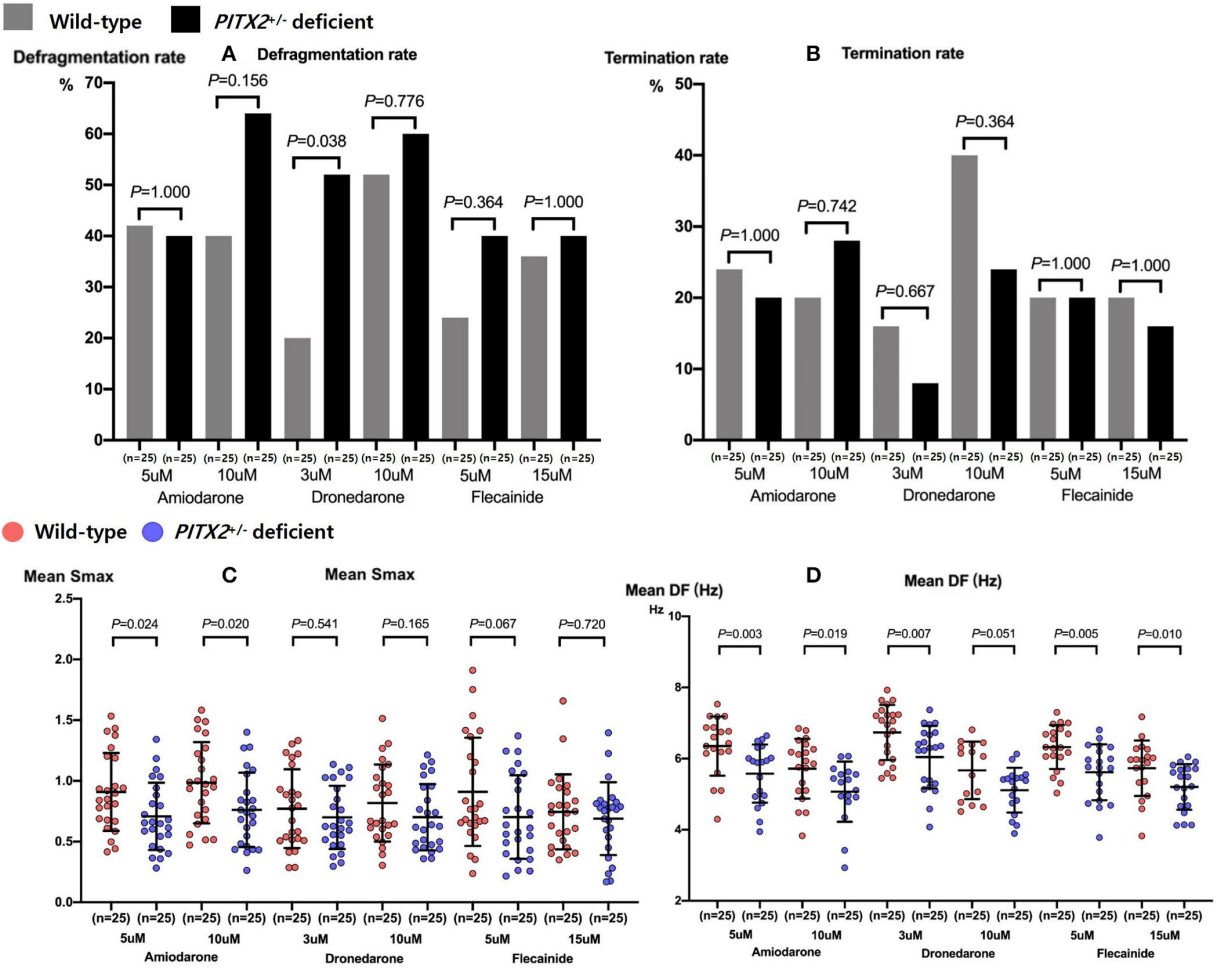
Genotype-dependent comparisons of the AF defragmentation **(A)** and AF termination **(B)** rates, mean Smax **(C)**, and DF **(D)** depending on the high and low doses of the three types of AADs. Every group includes identical number of samples (*n* = 25). Smax indicates the maximal slope of the restitution curves; DF, Dominant frequency; *PITX2*, paired-like homeodomain transcription factor 2.

We compared the genotype-dependent comparisons of the AF defragmentation and termination rates and mean DF and Smax depending on the AADs and their dosages (Figure 4). There was a significant difference in the genotype-dependent AF defragmentation rate with low dose dronedarone (*p* = 0.038, Figures 4A,B). The post-AAD mean DF was significantly lower under the *PITX2*^+/−^ deficient condition than in the wild type (*p* < 0.001, Table 2). The post-amiodarone Smax was significantly lower in the *PITX2*^+/−^ deficient condition than wild type (low dose *p* = 0.024; high dose *p* = 0.02), but not with dronedarone or flecainide (Figure 4C).

### *PITX2^+/−^* genotype-dependent clinical outcomes

We calculated the weighted genetic risk score (wGRS) in all 25 patients by multiplying the number of AF risk alleles by the beta coefficient for each single nucleotide polymorphism (SNP) and adding them (rs2595107, rs2200733, rs6843082, and rs10033464) together (Table 3). The 1- and 2-year clinical AF recurrence rates were compared depending on the *PITX2*^+/−^ wGRS. Although the patients with a higher wGRS tended to have a higher one-year AF recurrence, it was not statistically significant (*p* = 0.342, Log-rank *p* = 0.273, Supplementary Figure 1A). All 25 patients were one-AAD resistant (*n* = 22), two-AAD resistant (*n* = 1), or AAD-intolerable (*n* = 2) patients (Supplementary Table 1). We could not compare the genetic effects on the AAD responsiveness because of an AAD selection bias in the clinical setting.

**Table 3:** Clinical AF recurrence based on the *PITX*2^+/−^ risk score.

	***PITX*2^+/−^risk score**	***PITX*2^+/−^risk score**	***PITX*2^+/−^risk score**	***PITX*2^+/−^risk score**	***P*-value**	**Log rank P**
	**(0~6, *n* = 25)**	**(0~3, *n* = 7)**	**(4, *n* = 8)**	**(5~6, *n* = 10)**		
1 year recurrence	36.00%	14.30%	37.50%	50.00%	0.342	0.273
	(9/25)	(1/7)	(3/8)	(5/10)		
2 year recurrence	48.00%	42.90%	37.50%	60.00%	0.687	0.441
	(12/25)	(3/7)	(3/8)	(6/10)		

*AF, atrial fibrillation; PITX2^+/−^ risk score: Paired-like homeodomain transcription factor 2 (PITX2) gene risk score, calculated by multiplying the number of AF risk alleles by the beta coefficient for each single nucleotide polymorphism (SNP), and adding them (rs2595107, rs2200733, rs6843082, and rs10033464) together*.

## Discussions

### Main findings

In this study, we explored the anti-AF effects of virtual AF ablation and AADs according to the genotypes using realistic human AF computational modeling. Virtual AF ablations resulted in a higher AF defragmentation rate than virtual AADs in the overall AF episodes. Comparing the *PITX*2^+/−^ deficient and wild-type AF types, the AF defragmentation rate did not differ depending on the genotype after a virtual AF ablation. With consistency, the genetic risk score of the *PITX2*^+/−^ patients did not affect the rhythm outcome of the AF ablation in the clinical condition. However, *PITX*2^+/−^ deficient AF was more easily defragmented with a lower mean DF and Smax than the wild type after virtual AADs. Therefore, consistent with the previous clinical studies, virtual AF ablation exhibited an effective anti-AF effect regardless of the *PITX2* genotype, whereas virtual AADs exhibited more significant defragmentation or wave-dynamics change in the *PITX*2^+/−^ deficient genotype.

### Electrophysiological characteristics of *PITX2^+/−^* deficient AF

Genome-wide association studies (GWASs) have identified a number of SNPs that are associated with AF (5). Some SNPs located on chromosome 4q25 specifically increase AF susceptibility by modulating the activity of paired-like homeodomain transcription factor 2 (*PITX2*) in European, Japanese, Korean, and multi-ethnic populations with consistency (30). In the experimental models, variants in the *PITX2* gene create AF vulnerable conditions by changing the electrophysiological characteristics. The *PITX2*^+/−^ deficient murine atrial model exhibited a slightly depolarized resting membrane potential, reduced APD and AP amplitude (13), and low-voltage P waves and irregular beats, which indicated an impaired atrial conduction (31). The *PITX2*^+/−^ deficiency is related to triggered activity caused by abnormal calcium management (32) and provokes AF by causing a modification of the calcium handling and cell-cell communication. In this study, we applied the electrophysiological characteristics of the *PITX2* variant known by previous experimental studies to realistic computational modeling and generated a tissue or organ level *PITX2*^+/−^ deficient condition. In addition, we tested multiple virtual interventions under the same conditions with very high-resolution wave-dynamics parameters that are difficult to compare with clinical or experimental studies using computational modeling (33).

### Comparisons of the clinical studies and modeling studies on *PITX2^+/−^* deficient AF

There have been multiple clinical studies regarding the genotype-specific responsiveness of AF treatment. In particular, there is controversy about the effect of the *PITX2* variant on AF recurrence after AFCA. Husser et al. and Shoemaker et al. reported that the recurrence rate after AFCA was significantly higher in *PITX2* variants, especially rs2200733 (6–8), but the Korean AF Network registry study, which includes the highest number of patients, did not show any genotype-dependent differences after AFCA (9). Although the reason is not clear, ethnic differences may exist in the frequency of AF-related SNPs.

Parvez et al. reported a higher recurrence of AF after electrical cardioversion in patients with *PITX2* rs2200733 variants and 55% of the included patients were under AADs (11). They also reported that variants of rs10033464 at the *PITX2* gene were independent predictors of a successful AF rhythm control by AADs (12). Bai et al. and Syeda et al. reported that the class I AAD flecainide was more effective in suppressing atrial arrhythmias in *PITX2 v*ariants than in the wild type (13, 17). In contrast, Holmes et al. reported that the class III AAD dronedarone offered a more prominent anti-AF effect than flecainide or propafenone in a murine *PITX2*^+/−^ heart model than in the wild type (10). In this study, we confirmed that class I AAD was more effective in *PITX2* variants, consistent with the previous studies by Bai or Syeda (13, 17). In addition, we found the differences in AF wave dynamics and effects under class III AADs according to the *PITX2* genotype. However, there was no significant difference after AF ablation.

In this modeling study, virtual AF ablation tended to have a lower defragmentation rate in the *PITX*2^+/−^ deficient condition than in the wild type without a statistical significance. With consistency, clinical recurrence of AF after clinical AF ablation tended to be higher in patients with a high genetic risk score of the *PITX2*^+/−^ without statistical significance. The AF defragmentation rate was significantly higher in the *PITX*2^+/−^ deficient patients than in the wild-type patients after a virtual AAD administration.

### Potential role of computational modeling in AF management

Since Moe et al. presented the first human AF computational modeling (34), various atrial modeling approaches have been developed, with advancements in both higher-dimensional and realistic geometry models (14). The advantages of AF computational modeling include a high-density entire chamber map, reproducible condition control, virtual intervention trials, and prediction of the clinical outcome (33). With the development of computational technology, AF modeling has come to a point where it can be used in clinical AF treatment based on precision medicine. Boyle et al. have presented a clinically applicable rotor map as a proof of concept study by applying fibrosis reflected by cardiac MRI late gadolinium enhancement to AF computational modeling (35). We also developed realistic AF computational modeling (36) while considering the patient anatomy (cardiac computed tomogram), electrophysiology (3D-electroanatomical map), fibrosis (voltage map), and fiber orientation (LAT map) (16). By utilizing this realistic AF modeling (CUVIA, Laon Med Inc.), Kim et al. (37) and Baek et al. (38, 39) reported an improved rhythm outcome after modeling-guided linear ablation or DF ablation compared to an empirical AF ablation by multi-center randomized clinical trials. In this study, we showed that the effects of virtual ablation or virtual AADs according to the genotype can be evaluated by utilizing AF computational modeling based on the AF wave-dynamics mechanism.

### Limitations

This study had some limitations in the computational simulations. First, the right atrium was not incorporated in the personalized modeling because it is not possible to define interatrial connections using the current image resolution. Second, the LA wall thickness was not implemented in the 3D LA model. Third, it was not clear whether the atrial fibrosis area obtained using a bipolar voltage map reflected the pathological replacement fibrosis. Fourth, we utilized the monolayer in the 3D LA model, but not multi-layers that could perform as endocardial and epicardial layers.

## Conclusion

Consistent with the previous clinical studies, the virtual CPVI had effective anti-AF effects regardless of the *PITX2* genotype, whereas virtual AADs exhibited more significant defragmentation or wave-dynamic changes in the *PITX*2^+/−^ deficient genotype.

## Data availability statement

The original contributions presented in the study are included in the article/supplementary material, further inquiries can be directed to the corresponding author/s.

## Ethics statement

The studies involving human participants were reviewed and approved by Institutional Review Board of Severance Cardiovascular Hospital, Yonsei University Health System. The patients/participants provided their written informed consent to participate in this study.

## Author contributions

ZJ contributed to the data, statistical analyses, and writing of the manuscript. IH contributed to the statistical analyses and data acquisition. O-SK contributed to the software programming and data acquisition. BL confirmed the data acquisition and references. J-WP contributed to the clinical data acquisition. H-TY, T-HK, BJ, and M-HL contributed to the clinical data acquisition and interpretation of clinical data. H-NP contributed to the study design, clinical data acquisition, data interpretation, and revision of the manuscript. All authors contributed to the article and approved the submitted version.

## Funding

This work was supported by a grant [HI21C0011] from the Ministry of Health and Welfare, a grant [NRF-2020R1A2B5B01001695] from the Basic Science Research Program run by the National Research Foundation of Korea (NRF) which is funded by the Ministry of Science, ICT & Future Planning (MSIP), and a grant [RS-2022-00141473] from Cross-ministerial tasks.

## Conflict of interest

The authors declare that the research was conducted in the absence of any commercial or financial relationships that could be construed as a potential conflict of interest.

## Publisher's note

All claims expressed in this article are solely those of the authors and do not necessarily represent those of their affiliated organizations, or those of the publisher, the editors and the reviewers. Any product that may be evaluated in this article, or claim that may be made by its manufacturer, is not guaranteed or endorsed by the publisher.

## Supplementary material

Supplementary Figure 1 Association of AF recurrence with the *PITX2* genetic risk score. **(A)** 1-year AF recurrence and *PITX2* risk score. **(B)** 2-year AF recurrence and *PITX2* risk score. AF indicates atrial fibrillation; *PITX2* risk score, Paired-like homeodomain transcription factor 2 (*PITX2*) gene risk score, calculated by multiplying the number of AF risk alleles by the beta coefficient for each single nucleotide polymorphism (SNP), and adding them (rs2595107, rs2200733, rs6843082, and rs10033464) together.

Supplementary Table 1 Baseline characteristics of the patients.

Supplementary Table 2 AAD ion current setting for the wild-type and *PITX*2^+/−^ deficiency.

Supplementary Table 3 References for the atrial cell ion currents depending on the AADs.

## Abbreviations

AAD: antiarrhythmic drug
AF: atrial fibrillation
AFCA: atrial fibrillation catheter ablation
AFCL: atrial fibrillation cycle length
APD_90_: action potential duration at 90% repolarization
AT: atrial tachycardia
CPVI: circumferential pulmonary vein isolation
CV: conduction velocity
DF: dominant frequency
IRQ: interquartile range
*PITX2*: paired-like homeodomain transcription factor 2
LA: left atrium
LAT: local activation time
Smax: maximal slope of action potential duration restitution curve
SR: sinus rhythm.
