# Supplementary figures and images for: Ablation and antiarrhythmic drug effects on PITX2+/− deficient atrial fibrillation: A computational modeling study
Source: Front Cardiovasc Med. 2022 Jul 19;9:942998. doi: 10.3389/fcvm.2022.942998 (PMC9343754; doi:10.3389/fcvm.2022.942998)

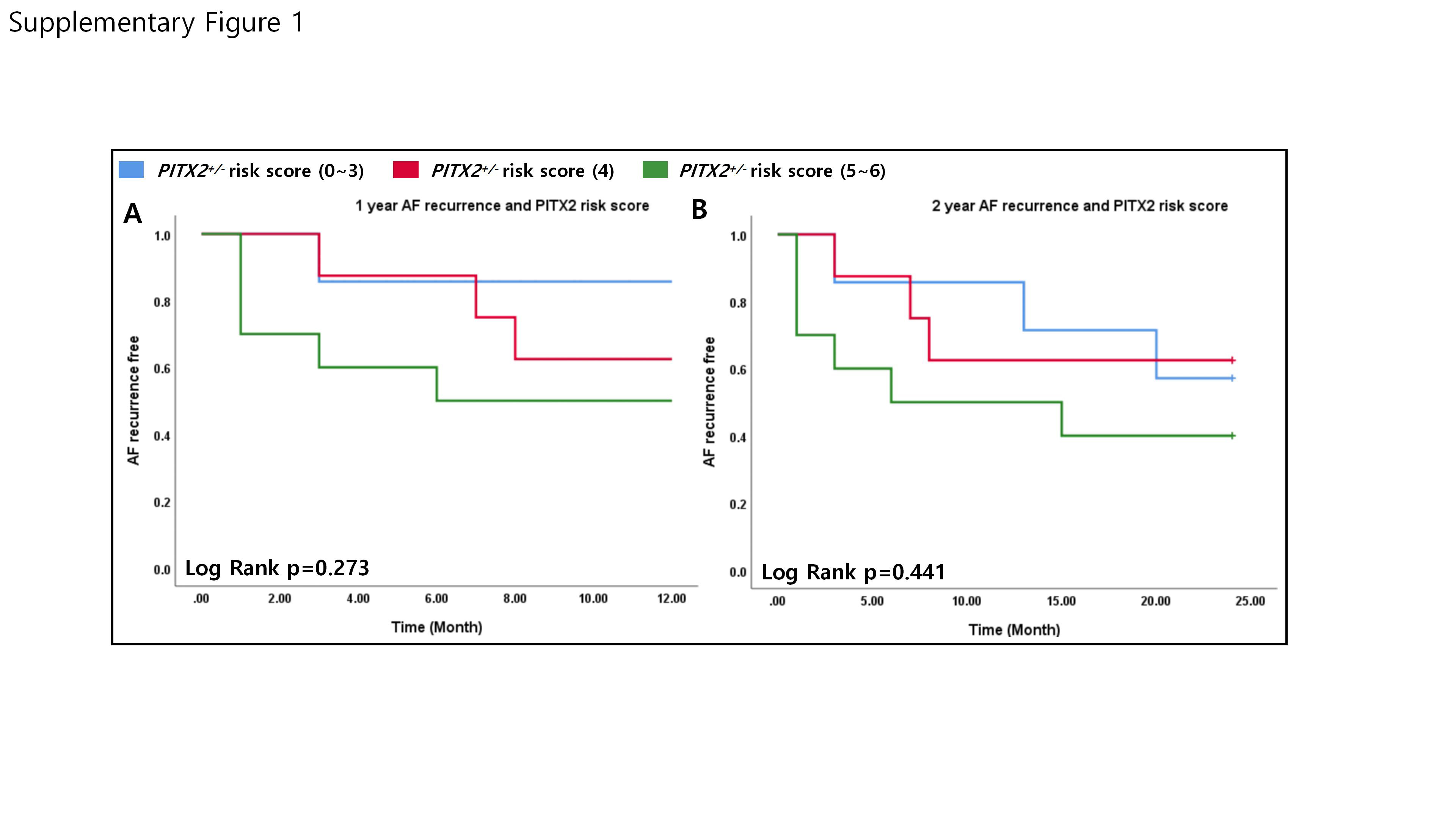

Supplement: Supplementary Figure 1 — Association of AF recurrence with the PITX2 genetic risk score. (A) 1-year AF recurrence and PITX2 risk score. (B) 2-year AF recurrence and PITX2 risk score. AF indicates atrial fibrillation; PITX2 risk score, Paired-like homeodomain transcription factor 2 (PITX2) gene risk score, calculated by multiplying the number of AF risk alleles by the beta coefficient for each single nucleotide polymorphism (SNP), and adding them (rs2595107, rs2200733, rs6843082, and rs10033464) together. [file Image_1.jpeg]
