# Supplementary material for: Ablation and antiarrhythmic drug effects on PITX2+/− deficient atrial fibrillation: A computational modeling study
Source: Front Cardiovasc Med. 2022 Jul 19;9:942998. doi: 10.3389/fcvm.2022.942998 (PMC9343754; doi:10.3389/fcvm.2022.942998)
Supplement: Supplementary Table 1 — Baseline characteristics of the patients. [file Data_Sheet_1.pdf]

**Supplementary Table 1. Baseline characteristics of the patients**

| Characteristics | |
|---|---|
| Male, n (%, n) | 68.0% (17/25) |
| Age, (Years) | 59.8±9.8 |
| <65, n (%, n) | 56.0% (14/25) |
| 65–74, n (%, n) | 40.0% (10/25) |
| ≥75, n (%, n) | 4.0% (1/25) |
| Paroxysmal AF, n (%, n) | 32.0% (8/25) |
| Follow-up Duration, (Months) | 14.2±15.3 |
| BMI, ($Kg/m^2$) | 24.6±2.9 |
| $CHA_2DS_2$-VASc Score | 2.0±1.4 |
| Heart failure, n (%, n) | 12.0% (3/25) |
| Hypertension, n (%, n) | 44.0% (11/25) |
| Diabetes, n (%, n) | 16.0% (4/25) |
| Stroke/TIA, n (%, n) | 20.0% (5/25) |
| Vascular Disease, n (%, n) | 8.0% (2/25) |
| Echocardiographic Parameters | |
| LA Dimension, (mm) | 42.6±6.1 |
| LA Volume Index, ($mL/m^2$) | 40.4±9.2 |
| LVEF (%) | 63.6±6.8 |
| E/Em | 10.6±3.8 |
| LA Voltage, (mV) | 1.9±0.8 |
| Class IC AAD resistant | |
| *PITX2*$^{+/-}$ risk score (0~6) (%, n) | 36.0% (9/25) |
| *PITX2*$^{+/-}$ risk score (0~3) (%, n) | 42.9% (3/7) |
| *PITX2*$^{+/-}$ risk score (4) (%, n) | 25.0% (2/8) |
| *PITX2*$^{+/-}$ risk score (5~6) (%, n) | 40.0% (4/10) |
| Class III AAD resistant | |
| *PITX2*$^{+/-}$ risk score (0~6) (%, n) | 60.0% (15/25) |
| *PITX2*$^{+/-}$ risk score (0~3) (%, n) | 42.9% (3/7) |
| *PITX2*$^{+/-}$ risk score (4) (%, n) | 62.5% (5/8) |
| *PITX2*$^{+/-}$ risk score (5~6) (%, n) | 70.0% (7/10) |

BMI: Body Mass Index, TIA: Transient Ischemic Attack, LVEF: Left Ventricular Ejection Fraction, E: Early Diastolic Transmitral Flow Velocity, Em: Early Diastolic Mitral Annular Velocity: AAD, antiarrhythmia drug, *PITX2*$^{+/-}$ risk score: Paired-like homeodomain transcription factor 2 (*PITX2*) gene risk score, calculated by multiplying the number of AF risk alleles by the beta coefficient for each single nucleotide polymorphism (SNP), and adding them (rs2595107, rs2200733, rs6843082 and rs10033464) together.

**Supplementary Table 2. AAD ion current setting for the Wild-type and *PITX2*$^{+/-}$ deficiency**

| | Wild-type AF | | | | | | |
|---|---|---|---|---|---|---|---|
| | **Baseline** | **Amiodarone 5 uM (%)** | **Amiodarone 10 uM (%)** | **Dronedarone 3 uM (%)** | **Dronedarone 10 uM (%)** | **Flecainide 5 uM (%)** | **Flecainide 15 uM (%)** |
| **gNa** | 90 | 90 | 85 | 90 | 81 | 75 | 45 |
| **gK1** | 210 | 180 | 160 | 200 | 160 | 210 | 210 |
| **gto** | 30 | 30 | 30 | 30 | 30 | 30 | 19 |
| **gKr** | 100 | 85 | 75 | 85 | 68 | 100 | 100 |
| **gCaL** | 30 | 15 | 12 | 12 | 8 | 30 | 21 |
| **gKur** | 50 | 50 | 50 | 50 | 50 | 20 | 10 |
| **gKs** | 100 | 90 | 80 | 80 | 60 | 100 | 100 |
| **INaCa (Max)** | 100 | 100 | 100 | 100 | 100 | 100 | 100 |
| **INaK (Max)** | 100 | 100 | 100 | 100 | 100 | 100 | 100 |
| **Iup (Max)** | 100 | 100 | 100 | 100 | 100 | 100 | 100 |
| **Krel** | 100 | 100 | 100 | 100 | 100 | 100 | 100 |
| **Caup (Max)** | 80 | 80 | 80 | 80 | 80 | 80 | 80 |
| **Ach** | 100 | 22 | 15 | 100 | 100 | 100 | 100 |

| | *PITX2*$^{+/-}$ Deficiency AF | | | | | | |
|---|---|---|---|---|---|---|---|
| | **Baseline** | **Amiodarone 5 uM (%)** | **Amiodarone 10 uM (%)** | **Dronedarone 3 uM (%)** | **Dronedarone 10 uM (%)** | **Flecainide 5 uM (%)** | **Flecainide 15 uM (%)** |
| **gNa** | 90 | 90 | 85 | 90 | 81 | 75 | 45 |
| **gK1** | 158 | 135 | 120 | 150 | 120 | 158 | 158 |
| **gto** | 30 | 30 | 30 | 30 | 30 | 30 | 19 |
| **gKr** | 200 | 170 | 150 | 170 | 136 | 200 | 200 |
| **gCaL** | 30 | 15 | 12 | 12 | 8 | 30 | 21 |
| **gKur** | 50 | 50 | 50 | 50 | 50 | 20 | 10 |
| **gKs** | 100 | 90 | 80 | 80 | 60 | 100 | 100 |
| **INaCa (Max)** | 100 | 100 | 100 | 100 | 100 | 100 | 100 |
| **INaK (Max)** | 100 | 100 | 100 | 100 | 100 | 100 | 100 |

| | | | | | | | |
|---|---|---|---|---|---|---|---|
| **Iup (Max)** | 100 | 100 | 100 | 100 | 100 | 100 | 100 |
| **Krel** | 100 | 100 | 100 | 100 | 100 | 100 | 100 |
| **Caup (Max)** | 80 | 80 | 80 | 80 | 80 | 80 | 80 |
| **Ach** | 100 | 22 | 15 | 100 | 100 | 100 | 100 |

**Supplementary Table 3. References for the atrial cell ion currents depending on the AADs**

| AADs | Reference | Animal/human model | Method | Ion current change |
|---|---|---|---|---|
| **Amiodarone**<br>(5 μM, 10 μM)<br>(1-4) | Nathalie Lalevée et al., 2003<br>M Nishimura et al., 1989<br>Chinmay Patel et al., 2009<br>K Kamiya et al., 2001 | Human cardiomyocytes<br>Guinea pig cardiomyocyte<br><br>Rabbit ventricular myocytes | Microelectrode recording and patch-clamp<br>The single-pipette, whole-cell, voltage-clamp | gK1, gNa, gKr, gCaL, gKs, Ach |
| **Dronedarone**<br>(3 μM, 10 μM)<br>(5-7) | Chen KH. et al., 2016<br>Gautier P. et al., 2003<br>A Varró et al., 2001 | Rat cardiomyocytes<br>Guinea pig ventricular cardiomyocyte<br>Dog cardiomyocyte | Whole-cell, perforated patch voltage-clamp | gCaL, gKs, gNa, gK1, gKr |
| **Flecainide**<br>(5 μM, 15 μM)<br>(8-12) | Wang Z. et al., 1993<br><br>Ging Kuo Wang et al., 2003<br>Joffrey Ducroq et al., 2007<br>F Scamps et al., 1989<br>L Yue et al., 1999 | Human pluripotent stem cell-derived ventricular cardiomyocyte<br>Rat<br>Rabbit cardiomyocyte<br>Frog ventricular cell<br>Dog cardiomyocyte | Whole-cell patch voltage clamp, microscope, and confocal laser-scanning unit | gNa,gKur,gNa,gto,gCaL |

**References**

1. Kamiya K, Nishiyama A, Yasui K, Hojo M, Sanguinetti MC, Kodama I. Short- and Long-Term Effects of Amiodarone on the Two Components of Cardiac Delayed Rectifier K(+) Current. *Circulation* (2001) 103(9):1317-24. Epub 2001/03/10. doi: 10.1161/01.cir.103.9.1317.

2. Lalevee N, Nargeot J, Barrere-Lemaire S, Gautier P, Richard S. Effects of Amiodarone and Dronedarone on Voltage-Dependent Sodium Current in Human Cardiomyocytes. *J Cardiovasc Electrophysiol* (2003) 14(8):885-90. Epub 2003/08/02. doi: 10.1046/j.1540-8167.2003.03064.x.

3. Nishimura M, Follmer CH, Singer DH. Amiodarone Blocks Calcium Current in Single Guinea Pig Ventricular Myocytes. *J Pharmacol Exp Ther* (1989) 251(2):650-9. Epub 1989/11/01.

4. Patel C, Yan GX, Kowey PR. Dronedarone. *Circulation* (2009) 120(7):636-44. Epub 2009/08/19. doi: 10.1161/CIRCULATIONAHA.109.858027.

5. Chen KH, Xu XH, Sun HY, Du XL, Liu H, Yang L, et al. Distinctive Property and Pharmacology of Voltage-Gated Sodium Current in Rat Atrial Vs Ventricular Myocytes. *Heart Rhythm* (2016) 13(3):762-70. Epub 2015/11/26. doi: 10.1016/j.hrthm.2015.11.022.

6. Gautier P, Guillemare E, Marion A, Bertrand JP, Tourneur Y, Nisato D. Electrophysiologic Characterization of Dronedarone in Guinea Pig Ventricular Cells. *J Cardiovasc Pharmacol* (2003) 41(2):191-202. Epub 2003/01/28. doi: 10.1097/00005344-200302000-00007.

7. Varro A, Takacs J, Nemeth M, Hala O, Virag L, Iost N, et al. Electrophysiological Effects of Dronedarone (Sr 33589), a Noniodinated Amiodarone Derivative in the Canine Heart: Comparison with Amiodarone. *Br J Pharmacol* (2001) 133(5):625-34. Epub 2001/06/29. doi: 10.1038/sj.bjp.0704106.

8. Ducroq J, Printemps R, Guilbot S, Gardette J, Salvetat C, Le Grand M. Action Potential Experiments Complete Herg Assay and Qt-Interval Measurements in Cardiac Preclinical Studies. *J Pharmacol Toxicol Methods* (2007) 56(2):159-70. Epub 2007/07/03. doi: 10.1016/j.vascn.2007.03.009.

9. Scamps F, Undrovinas A, Vassort G. Inhibition of Ica in Single Frog Cardiac Cells by Quinidine, Flecainide, Ethmozin, and Ethacizin. *Am J Physiol* (1989) 256(3 Pt 1):C549-59. Epub 1989/03/01. doi: 10.1152/ajpcell.1989.256.3.C549.

10. Wang GK, Russell C, Wang SY. State-Dependent Block of Wild-Type and Inactivation-Deficient Na+ Channels by Flecainide. *J Gen Physiol* (2003) 122(3):365-74. Epub 2003/08/13. doi: 10.1085/jgp.200308857.

11. Wang Z, Fermini B, Nattel S. Mechanism of Flecainide's Rate-Dependent Actions on Action Potential Duration in Canine Atrial Tissue. *J Pharmacol Exp Ther* (1993) 267(2):575-81. Epub 1993/11/01.

12. Yue L, Feng JL, Wang Z, Nattel S. Effects of Ambasilide, Quinidine, Flecainide and Verapamil on Ultra-Rapid Delayed Rectifier Potassium Currents in Canine Atrial Myocytes. *Cardiovasc Res* (2000) 46(1):151-61. Epub 2000/03/23. doi: 10.1016/s0008-6363(99)00430-7.
